# The association between overweight/obesity and double diabetes in adults with type 1 diabetes; a cross-sectional study

**DOI:** 10.1186/s12902-021-00851-1

**Published:** 2021-09-16

**Authors:** Nathan WP Cantley, Kathryn Lonnen, Ioannis Kyrou, Abd A Tahrani, Hassan Kahal

**Affiliations:** 1 Department of Clinical Biochemistry, Southmead Hospital, North Bristol NHS Trust, BS10 5NB Bristol, UK; 2 Department of Diabetes and Endocrinology, Southmead Hospital, North Bristol NHS Trust, BS10 5NB Bristol, UK; 3 Bristol Weight Management and Bariatric Service, Southmead Hospital, North Bristol NHS Trust, BS10 5NB Bristol, UK; 4 Centre for Sport, Exercise and Life Sciences, Research Institute for Health & Wellbeing, Coventry University, CV1 5FB Coventry, UK; 5 Warwickshire Institute for the Study of Diabetes, Endocrinology and Metabolism (WISDEM), University Hospitals Coventry and Warwickshire NHS Trust, CV2 2DX Coventry, UK; 6 Aston Medical School, College of Health and Life Sciences, Aston Medical Research Institute, Aston University, B4 7ET Birmingham, UK; 7 Warwick Medical School, University of Warwick, CV4 7AL Coventry, UK; 8 Department of Diabetes and Endocrinology, University Hospitals Birmingham NHS Foundation Trust, Birmingham, UK; 9 Institute of Metabolism and Systems Research, University of Birmingham, Birmingham, UK; 10 Centre for Endocrinology, Diabetes and Metabolism, Birmingham Health Partners, Birmingham, UK

**Keywords:** Type 1 Diabetes Mellitus, Obesity, Overweight, Double diabetes, Insulin resistance

## Abstract

**Background:**

Double Diabetes (DD), type 1 diabetes (T1DM) + insulin resistance (IR), is associated with increased risk of micro/macro-vascular complications and mortality. Obesity can contribute to the development of DD. This study explored the prevalence of overweight/obesity and their association with DD in adults with T1DM.

**Methods:**

Cross-sectional study of consecutive adults with T1DM attending diabetes clinics in a secondary care hospital (January-November 2019). Estimated glucose disposal rate (eGDR) was used as a marker of IR, and an eGDR < 8 was used to identify individuals with DD.

**Results:**

One hundred seven adults with T1DM were included; female/male: 51/56; age [median (inter-quartile range): 30.0 (23–51) years]; BMI 25.4 (22.8–30.0) kg/m^2^. Overweight/obesity prevalence was 57/107 (53.3 %) [overweight: 30/107 (28 %); obesity: 27/107 (25.2 %)]. Compared to those with normal BMI, individuals with T1DM and overweight/obesity had longer diabetes duration; higher total daily insulin dose; and higher DD prevalence: 48/57 (84.2 %) vs. 14/50 (28 %) (p < 0.01); with similar HbA1c. BMI correlated with total daily insulin dose (rho = 0.55; p < 0.01). Individuals with DD were older, had longer duration of diabetes, higher HbA1c, and more adverse lipid profile and microalbuminuria compared to those without DD.

**Conclusions:**

Overweight/obesity is very common in adults with T1DM, and is associated with double diabetes. BMI is positively associated with total insulin dose. Double diabetes is associated with adverse cardiovascular risk profile and is also common in lean individuals with T1DM. Further research is needed to examine the impact of overweight/obesity in people with T1DM and whether weight loss in this population can improve diabetes-related outcomes.

## Background

Obesity is a chronic disease that is closely associated with insulin resistance and significant morbidity [1, 2]. The prevalence of obesity globally has tripled between 1975 and 2016 [3]. In England, the 2017 Health Survey (HSE) revealed that 64.3 % of adults are overweight (35.6 %) or have obesity (28.7 %) [4].

Type 2 Diabetes mellitus (T2DM) is typically linked to obesity with 90 % of people with T2DM having a body mass index (BMI) in the overweight or obesity range [5]. In contrast, individuals with Type 1 diabetes mellitus (T1DM) are generally lean at diagnosis [6]. However, studies from North America suggest an increasing prevalence of overweight and obesity in people with T1DM [6, 7]. Of note, in the Pittsburgh Epidemiology of Diabetes Complications study, 42 % of adults with T1DM were overweight and 22.7 % had obesity after 18 years of follow up [6–8]. Despite such studies, data on the prevalence of overweight and obesity in individuals with T1DM from other populations/cohorts remain limited. Interestingly a recent review suggested that obesity may be a contributing factor to the increasing incidence of T1DM [9].

Double diabetes (DD) is a term introduced to describe individuals with T1DM who also have features of insulin resistance [10]. Whilst there have been different definitions for DD, estimated glucose disposal rate (eGDR) has recently been proposed as a good marker of insulin resistance that is easy to measure in clinical practice [10]. As such, a cut-off eGDR value of < 8 in people with T1DM has been proposed to identify individuals with DD [10]. DD is associated with increased risk of micro- and macrovascular complications, as well as higher cardiovascular disease (CVD) and all-cause mortality [10, 11].

Given the paucity of relevant data, this study aimed to examine the prevalence of overweight and obesity in adult individuals with T1DM. A secondary study aim was to examine the associations between overweight/obesity and DD in this population.

## Methods

We conducted a cross-sectional study examining consecutive adults with T1DM who attended the Young Adult Diabetes Clinic (individuals with age between 18 and 25 years) and the General Adult Diabetes Clinic (individuals > 25 years old) between 1st January and 25th November 2019 at Southmead Hospital, North Bristol NHS Trust, Bristol, UK. All investigations were performed as part of routine clinical care. The Patient Safety, Assurance and Audit (PSAA) committee at North Bristol NHS Trust (the ethics committee at the trust which approves clinical audits) approved this audit (audit number: CA8757). Consent was not needed as this was a retrospective audit performed as part of a service evaluation, rather than a research study – this was approved by the PSAA committee. Only clinically available data, as part of routine care, was included in the audit. The reporting of the study was carried out in accordance with STrengthening the Reporting of OBservational studies in Epidemiology (STROBE) guidelines on observational studies.

The T1DM diagnosis was determined based on the age of diagnosis, presence of T1DM-related autoantibodies (GAD, IA-2, and zinc 8 transporter), the measurement of urinary or blood c-peptide, and history of diabetic ketoacidosis in accordance with the Association of British Clinical Diabetologists (ABCD) criteria for diagnosing T1DM [12]. Participants were included in the study if they fulfilled any of the following criteria: (1) low urine or blood c-peptide, regardless of age of diagnosis; (2) two or more positive T1DM-related autoantibodies, regardless of age of diagnosis; (3) one positive T1DM-related autoantibody, and diagnosed < 30 years of age; (4) history of diabetic ketoacidosis (DKA), and diagnosed < 30 years of age; (5) diagnosed < 20 years of age, regardless of T1DM-related autoantibody status. Participants with T2DM, maturity onset diabetes of the young (MODY), diabetes secondary to underlying medical conditions (e.g. due to pancreatitis or steroid induced diabetes), and individuals where the diabetes diagnosis was not clear or yet confirmed were excluded from this study.

Overweight was defined as a BMI of 25-29.9 kg/m^2^, and obesity as a BMI ≥ 30 kg/m^2^. For people of Asian ethnic background, BMI cut-offs of 23-27.4 kg/m^2^ and ≥ 27.5 kg/m^2^ were used to identify people with overweight and obesity, respectively [13].

Participants’ data including height, weight, blood pressure, medications and past medical history, as well as biochemistry information including lipid profile and haemoglobin A1c (HbA1c – measured by high performance liquid chromatography) were collected from electronic Participants’ records. Hypertension was defined as recorded blood pressure > 140/90mmHg; use of blood pressure lowering medications; or a documented relevant diagnosis in the electronic Participant record [14]. Estimated glucose disposal rate (eGDR) was calculated as a validated proxy of insulin resistance based on the following formula: eGDR = 19.02 – (0.22 x BMI [kg/m^2^) – (3.26 x hypertension status [yes = 1, no = 0]) – (0.61 x HbA1c [converted from IFCC mmol/mol to NGSP %]) [10]. An eGDR value of < 8 was used to identify individuals with DD [10]. Data were collected, anonymised and stored on a secure NHS computer in line with data protection guidelines.

### Statistical analysis

Data are presented as median and interquartile range (IQ) or numbers (percentages), unless stated otherwise. Data were checked for normality using Histogram and Shapiro-Wilk test. Between groups comparisons, normal BMI vs. overweight/obesity, were assessed using the Mann-Whitney U test, or chi-square for frequencies. Correlations were assessed using Spearman rank-order correlation coefficient, as appropriate. A p-value of < 0.05, 2-tailed, was considered statistically significant. Data were analysed using PASW statistics 26 package (SPSS Inc., Chicago, USA) [15].

## Results

**Fig. 1:**
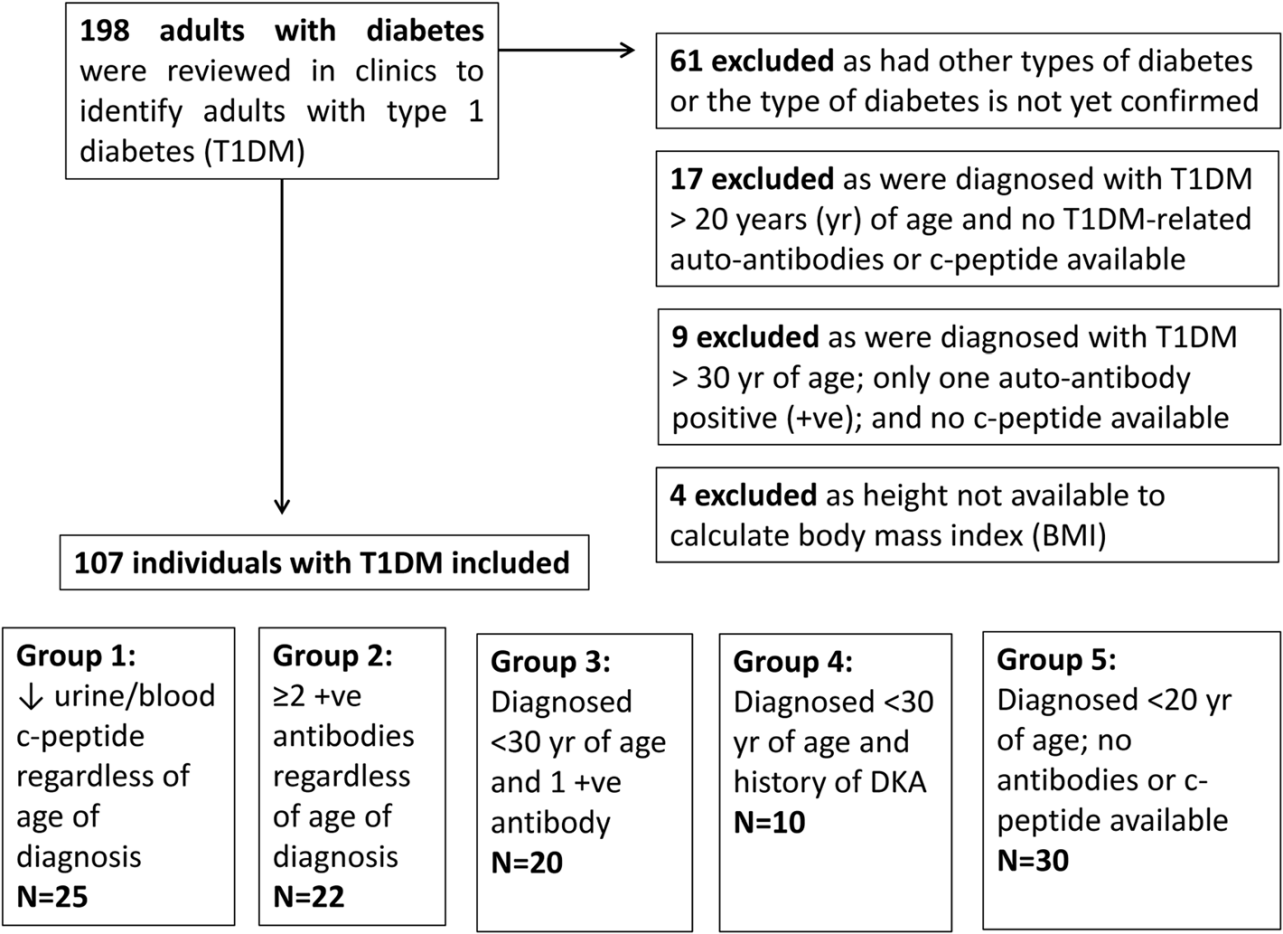
Flow chart of eligible study participants summarising the reasons for inclusion or exclusion. BMI: body mass index; DKA: diabetic ketoacidosis; yr: year; +ve: positive; ↓: low

In total, 198 individuals with diabetes were reviewed in the participating diabetes clinics during the study period. Of those, 107 were eligible for inclusion (Fig. 1). Table 1 summarizes demographics and pertinent clinical characteristics for the entire study cohort and by weight subgroup category (normal BMI, overweight, and obesity).

**Table 1:** Selected key study relevant demographic and clinical characteristics of the entire study cohort of adults with type 1 diabetes (T1DM), and by weight sub-group based on body mass index (BMI). Data are presented as median and interquartile range (IQ), and as percentages for frequencies. An estimated glucose disposal rate (eGDR) cut-off value of <8 was applied to identify individuals with double diabetes (T1DM combined with insulin resistance). Microalbuminuria was defined as urine albumin/creatinine ratio >3 mg/mmol. Hypertension was defined as blood pressure >140/90 mmHg or a documented relevant diagnosis in the electronic Participant record. ACEI, angiotensin-converting-enzyme inhibitors; ARB, Angiotensin II receptor blockers; BMI: body mass index; eGDR: estimated glucose disposal rate; HbA1c: haemoglobin A1c. *p-value <0.05 for overweight/obesity vs. normal BMI.

**Selected study relevant characteristics**	**Entire study cohort(*n*=107)**	**Sub-group**			
		**Normal BMI(*n*=50)**	**Overweight (*n*=30)**	**Obesity(*n*=27)**	**Overweight/obesity(*n*=57)**
Age at clinic (years)	30.0 (23.0-49.5)	26.0 (22.0-47.3)	30.5 (23.0-54.0)	38.0 (25.0-51.0)	34 (23.5-51.0)
Age at diagnosis (years)	17.0 (11.0-27.0)	18.0 (11.0-27.5)	18.0 (11.8-28.3)	15.0 (7.0-25.0)	17.0 (9.0-27.5)
Diabetes duration (years)	12.0 (5.5-23.5)	9.5 (4.0-17.0)	12.0 (7.5-25.0)	19.0 (10.0-31.0)	15.0 (9.0-27.0)*
Female [n (%)]	51 (48)	22 (44)	15 (50)	14 (52)	29 (51)
Body weight (kg)	74.3 (63.6-87.4)	63.8 (60.1 -73.7)	74.5 (68.5-81.9)	93.9 (87.4-111)	82.0 (74.0-95.7)*
BMI (kg/m^2^)	25.4 (22.8-29.8)	22.8 (20.6-23.9)	26.3 (25.8-27.7)	34.4 (31.2-37.8)	29.4 (26.1-34.3)*
HbA1c (mmol/mol)	70.0 (62.0-80.0)	70.0 (61.0-81.5)	70.5 (61.8-79.0)	70.0 (63.0-81.0)	70.0 (63-79.5)
Total Insulin dose (units)	49.0 (36.0-65.0)	42 (30.9-53.8)	51.5 (34.8-65.5)	78.0 (60.0-120.0)	60 (46.0-83.0)*
Insulin dose (unit/kg/day)	0.68 (0.53-0.89)	0.66 (0.48-0.76)	0.67 (0.51-0.86)	0.85 (0.56-1.1)	0.75 (0.56-0.95)*
Total Cholesterol (mmol/L)	4.3 (3.6-4.9)	4.2 (3.5-4.9)	4.4 (3.6-4.9)	4.4 (3.8-5.0)	4.4 (3.6-5.0)
Triglyceride (mmol/L)	1.1 (0.7–1.7)	1.0 (0.7-1.4)	1.2 (0.7-1.8)	1.3 (0.9-2.0)	1.3 (0.8-1.8)
HDL Cholesterol (mmol/L)	1.4 (1.2-1.8)	1.6 (1.3-1.9)	1.5 (1.2-1.9)	1.3 (1.1-1.5)	1.4 (1.1-1.7)*
LDL Cholesterol (mmol/L)	2.3 (1.7-2.9)	2.2 (1.6-2.6)	2.3 (2.0-3.0)	2.8 (1.8-3.4)	2.5 (1.9-3.1)
Hypertension, [n (%)]	20 (19)	4 (8)	7 (23)	9 (33)	16 (28)*
Statins [n (%)]	33 (31)	11 (22)	8 (27)	14 (52)	22 (39)
ACEI/ARB [n (%)]	22 (21)	4 (8)	6 (20)	12 (44)	18 (32)*
Metformin [n (%)]	9 (8)	2 (4)	1 (3)	6 (22)	7 (12)
Microalbuminuria, [n (%)]	17 (16)	5 (10)	5 (17)	7 (26)	12 (21)
eGDR	7.4 (5.7-8.7)	8.7 (7.4-9.7)	7.4 (6.0-8.2)	4.5 (3.7-6.8)	6.3 (4.5-7.6)*
Double diabetes, [n (%)]	62 (58)	14 (28)	21 (70)	27 (100)	48/57 (84)*

The study population was mainly of young adults with balanced numbers of men and women and long diabetes duration > 10 years. Ethnicity was documented in 72 participants (72/107; 67.3 %); of those with documented ethnicity, one Participant was of Asian ethnic background (1/72; 1.4 %), and the rest were White Caucasian (71/72; 98.6 %). Participants with overweight/obesity were older, had higher total daily insulin dose, and higher prevalence of hypertension compared to normal BMI. HbA1c and lipid profile were similar between Participants with normal weight and those with overweight/obesity.

The prevalence of overweight/obesity was 57/107 (53.3 %) [overweight: 30/107 (28 %); and obesity: 27/107 (25.2 %)]. A moderate association between increasing BMI and total daily insulin dose (rho = 0.55, p < 0.01) was noted (Table 2).

**Table 2:** Correlation between study pertinent variables by Spearman rank-order correlation coefficient (rho). BMI, body mass index; HbA1c, haemoglobin A1c. *P < 0.05 (2-tailed); **P < 0.01 (2-tailed)

	BMI (kg/m^2^)	Age at clinic (years)	Diabetes duration (years)	Total insulin dose (units)	HbA1c (mmol/mol)
**BMI (kg/m**^**2**^**)**		0.22*	0.27**	0.55**	0.12
**Age at clinic (years)**			0.44**	0.02	0.02
**Diabetes duration (years)**				0.18	0.01
**Total insulin dose (units)**					0.20*
**HbA1c (mmol/mol)**					

The prevalence of DD in the study cohort was 62/107 (57.9 %). There was a strong relationship between BMI and DD prevalence (Table 1), and all study Participants with T1DM and obesity had DD (27/27, 100 %). Individuals with DD were older, had longer duration of diabetes, higher HbA1c despite higher total insulin doses, more adverse lipid profile and more microalbuminuria compared to those without DD. A comparison between individuals with and without DD is summarised in Table 3. The relative risk (RR) of having DD in the presence of hypertension, elevated Hba1c (> 8 %, 65mmol/mol) or elevated BMI (> 25 kg/m^2^) was 2.07 (95 % CI: 1.67–2.57; *p* < 0.0001), 2.03 (95 % CI: 1.25–3.29; *p* = 0.0042) and 3.12 (95 % CI: 1.97–4.94; *p* < 0.0001), respectively.

**Table 3:** A comparison between individuals with and without double diabetes. An estimated glucose disposal rate (eGDR) cut-off value of < 8 was applied to identify individuals with Double Diabetes (T1DM combined with insulin resistance). Data are presented as median and interquartile range (IQ), and as percentages for frequencies. Microalbuminuria was defined as urine albumin/creatinine ratio > 3 mg/mmol. Hypertension was defined as blood pressure > 140/90 mmHg; taking medications for hypertension; or a documented relevant diagnosis in the electronic Participant record. ACEI, angiotensin-converting-enzyme inhibitors; ARB, Angiotensin II receptor blockers; BMI: body mass index; eGDR: estimated glucose disposal rate; HbA1c: haemoglobin A1c. *p-value < 0.05

Selected study cohort characteristics	Double Diabetes	
	No (*n* = 45)	Yes (*n* = 62)
Age at clinic (years)	24.0 (21.5–38.5)	37.0 (24.0-51.3)*
Age at diagnosis (years)	18.0 (11.0-25.5)	16.5 (9.0–29.0)
Diabetes duration (years)	9.0 (2.0–17.0)	14.0 (8.8–26.5)*
Female [n (%)]	22 (49)	29 (47)
Body weight (kg)	64.0 (61.1–74.3)	80.5 (73.1–94.2)*
BMI (kg/m^2^)	22.9 (20.7–24.3)	29.1 (25.3–34.1)*
HbA1c (mmol/mol)	63.0 (53.5–73.0)	72.5 (66.8–87.0)*
Total Insulin dose (units)	42.0 (30.8–54.5)	57.0 (42.3–80.5)*
Insulin dose (unit/kg/day)	0.65 (0.45–0.82)	0.71 (0.56–0.93)*
Total Cholesterol (mmol/L)	4.1 (3.5–4.9)	4.4 (3.6-5.0)
Triglyceride (mmol/L)	0.9 (0.6–1.3)	1.3 (0.9–1.8)*
HDL Cholesterol (mmol/L)	1.6 (1.3–1.9)	1.4 (1.1–1.7)*
LDL Cholesterol (mmol/L)	2.0 (1.6–2.5)	2.5 (1.8–3.2)
Hypertension, [n (%)]	0 (0)	20 (32)*
Statins [n (%)]	10 (22)	23 (37)
ACEI/ARB [n (%)]	3 (7)	19 (31)*
Metformin [n (%)]	5 (11)	9 (15)
Microalbuminuria, [n (%)]	2 (4.4)	15 (24)*
eGDR	8.9 (8.5–10.0)	6.1 (4.5–7.3)*

Surprisingly, a quarter of lean individuals with T1DM had evidence of DD. A comparison between lean individuals with and without DD is summarised in Table 4. Lean individuals with DD, compared to those without, took similar total daily insulin dose, but were older, had higher HbA1c, and more microalbuminuria (Table 4).

**Table 4:** A comparison between lean participants (normal BMI; *n* = 50) with and without double diabetes. Data are presented as median and interquartile range (IQ), and as percentages for frequencies. ACEI, angiotensin-converting-enzyme inhibitors; ARB, Angiotensin II receptor blockers; BMI: body mass index; eGDR: estimated glucose disposal rate; HbA1c: haemoglobin A1c. *p-value < 0.05

Selected study cohort characteristics	Double diabetes in participant with normal BMI	
	No (*n* = 36)	Yes (*n* = 14)
Age at clinic (years)	24.5 (21.0-35.8)	39.0 (23.0-63.8)*
Age at diagnosis (years)	18.0 (11.0-24.5)	17.0 (11.3–51.8)
Diabetes duration (years)	9.0 (2.0–17.0)	9.5 (5.8–18.8)
Female [n (%)]	16 (44)	6 (43)
Body weight (kg)	63.4 (60.3–73.1)	67.3 (59.6–75.0)
BMI (kg/m^2^)	22.6 (20.1–23.5)	23.2 (22.0-24.6)
HbA1c (mmol/mol)	65.0 (55.0-74.8)	88.5 (70.8-105.3)*
Total Insulin dose (units)	42.0 (30.6–50.3)	41.5 (31.5–58.3)
Insulin dose (unit/kg/day)	0.65 (0.47–0.77)	0.68 (0.50–0.76)
Total Cholesterol (mmol/L)	3.8 (3.4–4.9)	4.3 (3.7–5.4)
Triglyceride (mmol/L)	0.9 (0.7–1.3)	1.3 (0.8–1.6)
HDL Cholesterol (mmol/L)	1.6 (1.3–1.9)	1.6 (1.4-2.0)
LDL Cholesterol (mmol/L)	2.0 (1.6–2.5)	2.2 (1.9-3.0)
Hypertension, [n (%)]	0 (0)	4 (29)*
Statins [n (%)]	7 (19)	4 (29)
ACEI/ARB [n (%)]	2 (6)	2 (14)
Metformin [n (%)]	4 (11)	3 (21)
Microalbuminuria, [n (%)]	0 (0)	5 (36)*
eGDR	9.0 (8.6–10.0)	7.1 (5.6–7.4)*

## Discussion

This study highlights the high prevalence of overweight and obesity in adults with T1DM. The prevalence of overweight/obesity in our study sample (53.3 %, median age of 30 years) is close to the prevalence of overweight/obesity within the 25–34 year age group in the general population in England (54.0 %) [4]. Furthermore, the proportion of adults with T1DM who had obesity in our cohort (25.7 %) is similar to that reported by Conway et al. in North America (22.7 %) [7]. This suggests that people with T1DM are at similar risk to obesity as the general population. This requires more attention, and highlights the need for more research to better understand the clinical consequences of overweight/obesity in T1DM and the impact of treatment strategies.

In addition, we found an association between the duration of T1DM and overweight/obesity. This is probably caused by similar factors that contribute to weight gain with increasing age, including reduced basal metabolic rate, fat free mass, and physical activity with ageing; and to hormonal changes that occur during the ageing process [16]. Moreover, we also noted that people with T1DM and overweight/obesity required higher doses of insulin to achieve similar glycaemic control to those with normal BMI. As insulin treatment may promote weight gain in people with T2DM and T1DM [17, 18], this creates a vicious cycle in this population; where overweight/obesity will result in the need for more insulin to overcome insulin resistance, whilst injecting more insulin would promote weight gain. Subsequently, the relationship between insulin treatment and weight gain in people in T1DM is probably bi-directional, and adds further challenges to the management of diabetes in this population. Indeed, diabulimia, an eating disorder where a participant with T1DM does not inject insulin in order to lose weight, is a serious problem in those affected with significant risk of DKA and mortality [19]. Therefore, further research is needed to better understand the pathophysiology and consequences of overweight/obesity in people with T1DM [10, 20].

Overall, three quarters of adults with T1DM who were at least overweight in our study had DD. This finding was even stronger in the study participants with obesity, with all these individuals having DD. Interestingly, a quarter of lean adults with T1DM also had DD. This could be due to age, lean individuals with DD where older than lean individuals without DD, while total daily insulin dose doesn’t seem to be a factor. It could also be related to other unmeasured factors, for example, waist circumference, ethnicity, family history of insulin resistance, and physical activity. Notably, large observational studies suggest that individuals with DD have a higher risk of micro-/macro-vascular complications and mortality compared to people with T1DM without insulin resistance [10]. Our study also showed that DD was associated with microalbuminuria, hypertension and adverse lipid profile suggesting increased CVD risk. Subsequently, overweight/obesity is an extra burden on the health of people with T1DM, and, thus, interventions aiming to prevent weight gain and/or that promote weight loss might be beneficial in reducing the risk of diabetes complications in this population. However, there is a lack of data on interventions that are effective in promoting weight loss/weight maintenance in people with T1DM. Interventions such as very-low carbohydrate diet, glucagon-like peptide-1 (GLP-1) analogues, sodium–glucose cotransporter 2 (SGLT2) inhibitors, and bariatric surgery have been suggested by some research groups [21–26]. SGLT2 inhibitors and GLP-1 analogues are widely used in people with T2DM with favourable impact on weight [23]. In the DEPICT-1 study, the use of a SGLT2 inhibitor (Dapagliflozin 5.0 mg once a day) was accompanied by a 2.96 kg weight loss at 24 weeks compared to placebo in people with T1DM [25]. Similarly, in the Lira-1 trail the use of a GLP-1 analogue (Liraglutide 1.8 mg once a day) was associated with 6.8 kg weight loss compared to placebo in individuals with T1DM who were overweight [26]. However, the experience in using GLP-1 analogues in people with T1DM remains limited [23, 24]; while the use and licensing of SGLT2 inhibitors in individuals with T1DM have been restricted due to the increased risk of diabetic ketoacidosis [23, 24]. In this respect, our study highlights the need for more research to identify effective weight management interventions, including establishing if the above anti-hyperglycaemic agents could be safe and effective for people with T1DM and overweight/obesity. Indeed, such weight loss interventions/treatments may also contribute to reduce the chronic inflammation present in T1DM [27], which may be further exacerbated by obesity.

### Study Limitations

The findings should be considered within the context of the study limitations, including the cross sectional nature of the study that does not allow to ascertain causation, and the lack of power calculation. Due to the retrospective nature of the study, we did not have information on socio-economic, waist circumference, family history of insulin resistance, or physical activity. In addition, all patients were from a single centre, and people with type 1 diabetes on insulin pump therapy were not included. Subsequently, the generalisability of the study findings may have been affected by these limitations; however, the prevalence of obesity in our cohort of people with T1DM was similar to other observational studies from North America [7].

## Conclusions

Overweight/obesity is very common in adults with T1DM, and is associated with double diabetes. BMI is positively associated with total daily insulin dose in this population. Double diabetes is associated with adverse cardiovascular risk profile and is also common in lean individuals with T1DM. Further research is needed to examine the impact of overweight/obesity in people with T1DM and whether weight loss in this population can improve diabetes-related outcomes.

## Data Availability

The datasets used and analysed during the current study are available from the corresponding author on reasonable request.

## Declarations

**Ethics approval**

The study was part of an audit assessing the diabetes service in Southmead Hospital, North Bristol NHS Trust approved by the Patient Safety, Assurance and Audit (PSAA) committee at North Bristol NHS Trust (audit number: CA8757). The need for consent was also waived by the approving committee. The reporting of the study was carried out in accordance with “STrengthening the Reporting of OBservational studies in Epidemiology (STROBE)” guidelines on observational studies.

## References

[1] Kyle TK, Dhurandhar EJ, Allison DB (2016). Regarding Obesity as a Disease: Evolving Policies and Their Implications. Endocrinol Metab Clin North Am.

[2] Merger SR, Kerner W, Stadler M (2016). Prevalence and comorbidities of double diabetes. Diabetes Res Clin Pract.

[3] World Health Organisation. Fact Sheets – Obesity and overweight. *WHO* 2018. Accessed December 2020 URL: https://www.who.int/news-room/fact-sheets/detail/obesity-and-overweight

[4] NHS Digital Health Survey for England 2018: Adult and children overweight and obesity, *NHS* 2019. URL: https://digital.nhs.uk/data-and-information/publications/statistical/health-survey-for-england/2018/health-survey-for-england-2018-data-tables Accessed December 2020.

[5] Public Health England, Adults obesity and Type 2 Diabetes *PHE* 2014. URL: https://assets.publishing.service.gov.uk/government/uploads/system/uploads/attachment_data/file/338934/Adult_obesity_and_type_2_diabetes_.pdf Accessed December 2020.

[6] Nathan DM, Zinman B, Diabetes Control and Complications Trial/Epidemiology of Diabetes Interventions and Complications (DCCT/EDIC) Research Group (2009). Modern-day clinical course of type 1 diabetes mellitus after 30 years’ duration: the diabetes control and complications trial/epidemiology of diabetes interventions and complications and Pittsburgh epidemiology of diabetes complications experience (1983–2005). Arch Intern Med.

[7] Conway B, Miller RG, Costacou T (2010). Temporal patterns in overweight and obesity in Type 1 diabetes. Diabet Med.

[8] The DCCT Research Group (1987). Diabetes Control and Complications Trial (DCCT): Results of Feasibility Study. Diabetes Care.

[9] Buzzetti R, Zampetti S, Pozzilli P. Impact of obesity on the increasing incidence of type 1 diabetes. Diabetes Obes Metab. 2020 Jul;22(7):1009–1013. doi: 10.1111/dom.14022. 10.1111/dom.14022 32157790

[10] Kietsiriroje N, Pearson S, Campbell M, Ariëns RAS, Ajjan RA (2019). Double diabetes: A distinct high-risk group?. Diabetes Obes Metab.

[11] Nyström T, Holzmann MJ, Eliasson B, Svensson AM, Sartipy U (2018). Estimated glucose disposal rate predicts mortality in adults with type 1 diabetes. Diabetes Obes Metab.

[12] ABCD - Standards of Care for Management of Adults with Type 1 Diabetes 2020, ABCD 2020. https://abcd.care/resource/standards-care-management-adults-type-1-diabetes-2020 Accessed December 2020.

[13] WHO Expert Consultation. Appropriate body-mass index for Asian populations and its implications for policy and intervention strategies. Lancet 2004 Jan 10;363(9403):157 – 63. doi: 10.1016/S0140-6736(03)15268-3. 10.1016/S0140-6736(03)15268-3 14726171

[14] de Boer IH, Bangalore S, Benetos A (2017). Diabetes and hypertension: a position statement by the American Diabetes Association. Diabetes Care.

[15] SPSS Inc. Released 2009. PASW Statistics for Windows, Version 18.0. Chicago: SPSS Inc.

[16] Villareal DT, Apovian CM, Kushner RF, Klein S, American Society for Nutrition et al. Obesity in older adults: technical review and position statement of the American Society for Nutrition and NAASO, The Obesity Society. *Am J Clin Nutr.* 2005 Nov;82(5):923–34. doi: 10.1093/ajcn/82.5.923. 10.1093/ajcn/82.5.923 16280421

[17] Russell-Jones D, Khan R. Insulin-associated weight gain in diabetes–causes, effects and coping strategies. *Diabetes Obes Metab*. 2007 Nov;9(6):799–812. 10.1111/j.1463-1326.2006.00686.x 17924864

[18] Kyrou I, Kumar S (2010). Weight management in overweight and obese Participants with type 2 diabetes mellitus. Br J Diabetes Vasc Dis.

[19] Winston AP. Eating Disorders and Diabetes. *Curr Diab Reports*. 2020 Jun; 20(8):32. doi: 10.1007/s11892-020-01320-0. 10.1007/s11892-020-01320-0 32537669

[20] Cleland SJ, Fisher BM, Colhoun HM, Sattar N, Petrie JR (2013). Insulin resistance in type 1 diabetes: what is ‘double diabetes’ and what are the risks?. Diabetologia..

[21] Kirwan JP, Aminian A, Kashyap SR, Burguera B, Brethauer SA, et al. Bariatric Surgery in Obese Participants With Type 1 Diabetes. *Diabetes Care* Jun 2016, 39 (6) 941–948; DOI: 10.2337/dc15-2732. 10.2337/dc15-2732 PMC8310563 27222552

[22] Lennerz BS, Barton A, Bernstein RK, Dikeman RD, Diulus C, et al. Management of Type 1 Diabetes With a Very Low-Carbohydrate Diet Pediatrics. 2018;141(6):e20173349. doi: 10.1542/peds.2017-3349. 10.1542/peds.2017-3349 PMC6034614 29735574

[23] Wilding JPH. Medication use for the treatment of diabetes in obese individuals. Diabetologia. 2018 Feb;61(2):265–272. doi: 10.1007/s00125-017-4288-1. 10.1007/s00125-017-4288-1 PMC6448959 28501906

[24] Casu A, Bilal A, Pratley RE; Advancing Care for Type 1 Diabetes, Obesity Network (ACT1ON). Pharmacological therapies to address obesity in type 1 diabetes. Curr Opin Endocrinol Diabetes Obes. 2020;27(4):194–206. doi: 10.1097/MED.0000000000000555. 10.1097/MED.0000000000000555 32618631

[25] Dandona P, Mathieu C, Phillip M, Hansen L, Griffen SC, et al. DEPICT-1 Investigators. Efficacy and safety of dapagliflozin in patients with inadequately controlled type 1 diabetes (DEPICT-1): 24 week results from a multicentre, double-blind, phase 3, randomised controlled trial. Lancet Diabetes Endocrinol. 2017;5(11):864–876. doi: 10.1016/S2213-8587(17)30308-X. 10.1016/S2213-8587(17)30308-X 28919061

[26] Dejgaard TF, Frandsen CS, Hansen TS, Almdal T, Urhammer S, et al. Efficacy and safety of liraglutide for overweight adult patients with type 1 diabetes and insufficient glycaemic control (Lira-1): a randomised, double-blind, placebo-controlled trial. Lancet Diabetes Endocrinol. 2016 Mar;4(3):221–232. doi: 10.1016/S2213-8587(15)00436-2. 10.1016/S2213-8587(15)00436-2 26656289

[27] Pozzilli P, Maddaloni E, Buzzetti R. Combination immunotherapies for type 1 diabetes mellitus. Nat Rev Endocrinol. 2015 May;11(5):289–97. doi: 10.1038/nrendo.2015.8. 10.1038/nrendo.2015.8 25688000

